# Workplace violence and sleep disorders in emergency nurses: a multicenter cross-sectional study

**DOI:** 10.3389/fpubh.2026.1821926

**Published:** 2026-04-14

**Authors:** Chunmei Zhang, Ya Zhang, Kun Ling, Luying Zhong, Yongli Gao

**Affiliations:** 1 Department of Emergency Medicine, West China Hospital, Sichuan University/West China School of Nursing, Sichuan University, Chengdu, China; 2 Disaster Medical Center, Sichuan University, Chengdu, China; 3 Nursing Key Laboratory of Sichuan Province, Chengdu, China

**Keywords:** correlation, emergency department, nurses, sleep disorders, workplace violence

## Abstract

**Background:**

Emergency nurses are at high risk of experiencing workplace violence (WPV), a significant global issue. Violence negatively impacts nurses' sleep quality.

**Objective:**

To investigate the prevalence of workplace violence and sleep disorders among emergency department (ED) nurses, and to explore their interrelationship, thereby providing evidence for developing strategies to reduce WPV and improve sleep quality.

**Methods:**

A stratified cluster sampling method was used to recruit ED nurses from 30 hospitals across mainland China between December 26, 2023, and January 18, 2024. Data were collected using a self-designed demographic questionnaire, the Workplace Violence Questionnaire, and the Self-administered Sleep Questionnaire (SSQ). Pearson correlation analysis and multiple linear regression were employed to examine the relationship between WPV and sleep disorders.

**Results:**

A total of 1,540 ED nurses participated in the study. Approximately 60% (913) exhibited sleep disorders, and 85.0% (1,309) had experienced workplace violence within the past year. WPV was significantly correlated with sleep disorders (*r* = 0.295, *P* < 0.01). Multiple linear regression analysis revealed that psychological violence score, years of work experience, weekly working hours, night shift frequency, and marital status were independent predictors of sleep disorder severity.

**Conclusion:**

Emergency nurses face a high level of workplace violence, which significantly contributes to sleep disorders. Enhanced management strategies to prevent WPV and improve sleep quality are urgently needed.

## 1 Introduction

Workplace violence (WPV) refers to any verbal or physical acts, harassment, threats, intimidation, or other abusive or harmful behaviors occurring in the workplace (1). The healthcare sector is one of the most vulnerable industries globally, with studies reporting that 19.3%−36.4% of healthcare workers have experienced WPV (2). The emergency department (ED), due to its unique environment—characterized by critical patient conditions, emotional volatility, overcrowding, and high-pressure doctor-patient communication—is particularly prone to violent incidents (3–5). Systematic reviews indicate that up to 77% of ED staff have encountered WPV during their careers (6). Specific studies on emergency nurses report an overall exposure rate of 79.39%, with non-physical violence at 78.38% and physical violence at 39.65% (7). Additionally, lateral violence from supervisors or colleagues—such as verbal degradation or social exclusion—is also prevalent, with an incidence of approximately 33.8% (8). Thus, emergency nurses are a high-risk group, facing serious challenges to their occupational safety and mental health.

WPV not only threatens physical safety but also leads to long-term psychological and health consequences, including anxiety, depression, burnout, and sleep disorders (9–11). Sleep disorders, a major public health concern worldwide, are particularly common among shift-working nurses (12, 13). Due to irregular schedules and frequent night shifts, nurses often suffer from circadian rhythm disruption, difficulty falling asleep, and fragmented sleep. Studies show that about 68% of hospital nurses experience some degree of sleep disturbance, with night shift nurses reporting significantly poorer sleep quality than day shift workers (14, 15). Emergency nurses, who face both high night shift frequency and high exposure to violence, exhibit even higher rates of sleep disorders (16, 17).

Evidence confirms a significant association between WPV and poor sleep quality (18). Whether physical or psychological violence from patients/families or covert aggression from colleagues, such experiences can trigger persistent psychological stress, leading to hyperarousal, emotional exhaustion, and negative cognitive loops that disrupt normal sleep architecture (19, 20). Victims often report delayed sleep onset, frequent nighttime awakenings, and early morning awakening (21, 22). Chronic sleep deprivation may, in turn, exacerbate anxiety and depression, creating a vicious cycle of “violence, psychological stress, sleep disorder, burnout” (23). Therefore, addressing sleep disorders among emergency nurses is imperative.

Although existing research on nurse sleep quality has covered demographic and workload factors, systematic studies focusing on the impact of different types of WPV, particularly physical vs. psychological violence, on sleep disorders among this high-risk group remain limited. This study aims to conduct a large-scale, multicenter cross-sectional survey to reveal the current status of WPV exposure and its relationship with sleep disorders among Chinese emergency nurses, providing scientific evidence for targeted interventions to improve occupational health.

## 2 Methods

### 2.1 Study design and participants

This was a cross-sectional study conducted from December 26, 2023, to January 18, 2024. Using stratified cluster sampling method to selected 3 to 5 hospitals randomly from each of China's seven major geographic regions (North China, Northeast China, East China, Central China, South China, Southwest China, and Northwest China), emergency nurses from 30 hospitals were recruited. The inclusion and exclusion criteria were the same as those in previous study (24), the inclusion criteria were: ① licensed nurses in the emergency department and ② had ≥ 1 year of emergency work experience. The exclusion criteria were: ① had a history of psychiatric disorders; ② were nurses on advanced training; and ③ were nurses on sick leave and breastfeeding (≥1 month). Based on a significance level of 0.05 and 80% power, a sample size of 1,330 was calculated from preliminary pilot data (25, 26). This study strictly adhered to ethical guidelines and was approved by the Ethics Committee of West China Hospital, Sichuan University. The review lot number is [2024 (309)]. The research was conducted in accordance with the principles of the Declaration of Helsinki. All participants provided written informed consent and were fully informed of the study's purpose and privacy protection measures.

### 2.2 Instruments

#### 2.2.1 Demographic questionnaire

Collected data on gender, age, education level, years of experience, night shift patterns, income, marital status, and fertility status.

#### 2.2.2 Workplace violence scale

A four-item scale adapted from previous studies assessed physical and emotional abuse from patients/families and supervisors/colleagues over the past year (27–29). Responses were scored on a Likert scale (0 = never, 6 = daily). Higher mean scores indicated more frequent exposure to violence. The scale demonstrated good reliability in this study (Cronbach's α = 0.784).

#### 2.2.3 Self-administered sleep questionnaire (SSQ)

Used to assess sleep disorder status (30), evaluating three domains: sleep onset latency, sleep maintenance, and early morning awakening. Presence of any one symptom was defined as a sleep disorder. Each item is scored on a scale of 0 to 4, higher scores indicated greater severity. The SSQ showed good internal consistency (Cronbach's α = 0.809).

### 2.3 Data collection

Prior to the survey, one core coordinator per hospital was trained to ensure standardized understanding of key concepts, instructions, and explanations. An electronic questionnaire was distributed via *Wenjuanxing* (https://www.wjx.com), including a study introduction, informed consent, and the main survey. Access was granted only after participants read and signed the consent form, ensuring ethical compliance. All items were mandatory, and duplicate submissions from the same IP address were blocked to ensure data integrity and uniqueness. Invalid questionnaires with excessively short response time, regular or identical response patterns, and logical contradictions were excluded.

### 2.4 Statistical analysis

Data were analyzed using IBM SPSS Statistics 26.0. Continuous variables were described using the M ± SD, while categorical variables were described using the proportion. Pearson correlation analysis was used to examine associations between WPV and sleep disorders. Univariate analysis was first conducted to identify variables significantly associated with sleep disorder scores (*P* < 0.05); *t*-tests or ANOVA were used for categorical variables, while Pearson's correlation analysis was used for continuous variables, which were then entered into a multiple linear regression model to identify independent predictors. A two-tailed *P* < 0.05 was considered statistically significant.

## 3 Results

### 3.1 Demographic characteristics

A total of 1,550 eligible participants were enrolled in the study. Ten questionnaires were excluded due to regular response patterns, yielding 1,540 valid responses. The overall response rate was 100%, with a valid questionnaire rate of 99.35%. The majority were female (78.6%, *n* = 1,211), aged 20–39 years (85.5%), and held bachelor's degrees or higher (90%), for details, see our team's previous study (24).

### 3.2 Prevalence of workplace violence and sleep disorders

The mean sleep disorder score was **8.56** **±3.12**, with approximately **60% (913)** of nurses experiencing sleep disorders. The mean WPV score was **2.20** **±** **1.97**, and **85.0% (1,309)** had experienced WPV in the past year. Specifically, **61.3% (943)** experienced physical violence, and **84.4% (1,299)** experienced psychological violence (Tables 1, 2).

**Table 1:** Mean scores of workplace violence and sleep disorders (*n* = 1,540).

Variable	Dimension	Min	Max	Mean	SD
Workplace violence	Physical	0	6	0.76	0.98
	Psychological	0	6	1.44	1.17
	Total	0	12	2.2	1.97
Sleep disorders	Sleep onset	1	5	2.85	1.128
	Sleep maintenance	1	5	2.83	1.236
	Early morning awakening	1	5	2.88	1.298
	Total	3	15	8.56	3.12

**Table 2:** Analysis of sleep status and workplace violence prevalence (*n* = 1,540).

Variable	Category	*n*	%
Workplace violence	Physical violence from patients/families	918	59.6
	Psychological violence from patients/families	1,287	83.6
	Physical violence from supervisors/colleagues	406	26.4
	Psychological violence from supervisors/colleagues	712	46.2
	Any workplace violence (Total)	1,308	85
	Physical violence (Total)	943	61.3
	Psychological violence (Total)	1,299	84.4
Sleep onset latency	0–10 min	154	10
	11–30 min	507	32.9
	31–59 min	433	28.1
	1–2 h	305	19.8
	2 h	141	9.2
Sleep maintenance	Never (or almost never)	256	16.6
	A few times per year	405	26.3
	More than once per month	379	24.6
	More than once per week	345	22.4
	Almost every night	155	10.1
Early morning awakening	Never (or almost never)	280	18.2
	A few times per year	372	24.2
	More than once per month	340	22.1
	More than once per week	356	23.1
	Almost every night	192	12.5
Sleep disorders	Present	913	59.3
	Absent	627	40.7

### 3.3 Correlation between workplace violence and sleep disorders

Pearson correlation analysis showed a significant positive correlation between WPV and sleep disorders (*r* = 0.295, *P* < 0.01). All dimensions of WPV were significantly correlated with sleep disorder components (*P* < 0.01) (Table 3).

**Table 3:** Correlation between workplace violence and sleep disorders.

Variable	Physical violence	Psychological violence	Total WPV	Sleep onset	Sleep maintenance	Early awakening	Total sleep score
Physical violence	1						
Psychological violence	0.674^**^	1					
Total WPV	0.899^**^	0.929^**^	1				
Sleep onset	0.138^**^	0.161^**^	0.164^**^	1			
Sleep maintenance	0.230^**^	0.318^**^	0.303^**^	0.489^**^	1		
Early awakening	0.208^**^	0.295^**^	0.279^**^	0.409^**^	0.839^**^	1	
Total sleep score	0.228^**^	0.307^**^	0.295^**^	0.725^**^	0.922^**^	0.896^**^	1

^**^ *P* < 0.01.

### 3.4 Analysis results of factors influencing sleep disorders

A univariate screening was first conducted to identify potential predictors of sleep disorders. The total sleep disorder score was treated as the dependent variable, while general demographic characteristics and workplace violence exposure were included as independent variables in the analysis. All independent variables are classified based on the actual clinical conditions in Chinese hospitals.

#### 3.4.1 Univariate screening

The univariate analysis revealed that **age**, **marital status**, **fertility status**, **years of work experience**, and **weekly working hours** were significantly associated with sleep disorder scores (*P* < 0.05), indicating statistical significance. These variables were therefore selected for inclusion in the subsequent multiple linear regression model to further identify independent predictors (Table 4).

**Table 4:** Univariate analysis results for sleep disorder scores.

Variable	Category	M ±SD	*t*/*F*	*P*
Age group	20–29 years	7.93 ± 3.13	15.567	**<0.001**
	30–39 years	8.89 ± 3.03		
	40–49 years	9.34 ± 3.09		
	≥50 years	7.90 ± 3.18		
Gender	Male	8.32 ± 3.22	1.581	0.114
	Female	8.62 ± 3.09		
Marital status	Unmarried	8.26 ± 3.19	−2.791	**0.005**
	Married	8.72 ± 3.07		
Fertility status	Childless	8.26 ± 3.13	−3.479	**<0.001**
	Has children	8.81 ± 3.09		
Education level	Diploma	8.21 ± 3.22	−1.621	0.105
	Bachelor's or above	8.60 ± 3.10		
Professional title	Junior	8.30 ± 3.12	8.200	**<0.001**
	Intermediate	8.97 ± 3.08		
	Senior	8.41 ± 3.14		
Years of experience	≤5 years	7.80 ± 3.077	12.817	**<0.001**
	6–10 years	8.64 ± 3.038		
	11–15 years	9.21 ± 3.036		
	16–20 years	9.17 ± 3.067		
	>20 years	9.01 ± 3.269		
Weekly working hours	<40 h	8.07 ± 3.09	8.435	**<0.001**
	41–48 h	8.77 ± 3.07		
	49–58 h	9.11 ± 3.30		
	≥59 h	9.30 ± 3.17		
Night shift frequency (per month)	0 times	8.28 ± 2.96	6.318	**<0.001**
	1–4 times	7.93 ± 2.98		
	5–8 times	8.58 ± 3.10		
	≥9 times	8.96 ± 3.23		
Monthly income	<4,000 CNY	8.86 ± 3.51	0.764	0.549
	4,000–5,999 CNY	8.86 ± 3.40		
	6,000–7,999 CNY	8.66 ± 3.17		
	8,000–9,999 CNY	8.51 ± 3.03		
	≥10,000 CNY	8.46 ± 3.06		

CNY, Chinese Yuan. The bold values indicate that the results are statistically significant, *P* < 0.05.

#### 3.4.2 Multivariate analysis

The multiple linear regression analysis revealed that being married, holding a senior professional title, having longer work experience (6–10 years, 11–15 years, 16–20 years, and >20 years), working 41–48 h per week, having a high frequency of night shifts (≥9 times per month), and higher scores on psychological violence were significant influencing factors associated with increased sleep disorder scores among emergency nurses (Table 5).

**Table 5:** Multivariate linear regression analysis of factors influencing sleep disorder scores (*n* = 1,540).

Variable	Category	*B*	*SE*	β	*t*	*P*	95% *CI*
(Constant)	—	6.174	0.308	—	20.047	<0.001	[5.570, 6.779]
Age	20–29 years (Ref.)	—	—	—	—	—	—
	30–39 years	0.364	0.317	0.058	1.147	0.252	[−0.259, 0.987]
	40–49 years	0.543	0.538	0.056	1.01	0.312	[−0.512, 1.598]
	≥50 years	−0.705	0.766	−0.036	−0.92	0.358	[−2.208, 0.798]
Marital status	Unmarried (Ref.)	—	—	—	—	—	—
	Married	−0.556	0.267	−0.086	−2.081	**0.038**	[−1.080, −0.032]
Fertility status	Childless (Ref.)	—	—	—	—	—	—
	Has children	0.071	0.267	0.011	0.265	0.791	[−0.453, 0.595]
Professional title	Junior (Ref.)	—	—	—	—	—	—
	Intermediate	−0.164	0.198	−0.025	−0.829	0.407	[−0.553, 0.225]
	Senior	−1.197	0.459	−0.074	−2.605	**0.009**	[−2.097, −0.296]
Years of experience	≤5 years (Ref.)	—	—	—	—	—	—
	6–10 years	0.69	0.31	0.103	2.23	**0.026**	[0.083, 1.298]
	11–15 years	1.217	0.386	0.155	3.156	**0.002**	[0.460, 1.974]
	16–20 years	1.302	0.507	0.112	2.566	**0.010**	[0.306, 2.297]
	>20 years	2.07	0.636	0.188	3.255	**0.001**	[0.823, 3.318]
Weekly working hours	<40 h (Ref.)	—	—	—	—	—	—
	41–48 h	0.607	0.161	0.097	3.775	**<0.001**	[0.291, 0.922]
	49–58 h	0.515	0.295	0.045	1.747	0.081	[−0.063, 1.093]
	≥59 h	0.565	0.401	0.035	1.409	0.159	[−0.222, 1.352]
Night shift frequency (per month)	0 times (Ref.)	—	—	—	—	—	—
	1–4 times	−0.067	0.309	−0.008	−0.218	0.828	[−0.674, 0.540]
	5–8 times	0.541	0.276	0.086	1.96	0.050	[0.000, 1.082]
	≥9 times	0.649	0.287	0.095	2.26	**0.024**	[0.086, 1.212]
Workplace violence	Physical violence	0.115	0.103	0.036	1.112	0.266	[−0.088, 0.317]
	Psychological violence	0.675	0.087	0.252	7.742	**<0.001**	[0.504, 0.846]

The bold values indicate that the results are statistically significant, *P* < 0.05.

### 3.5 Multicollinearity diagnosis

The multicollinearity diagnostic results showed that all tolerance values >0.1 and all VIF < 10, indicating no significant multicollinearity among the independent variables.

## 4 Discussion

This multicenter cross-sectional study involving 1,540 emergency nurses from 30 hospitals across China revealed that workplace violence (WPV) is highly prevalent among emergency nursing staff and is significantly positively correlated with sleep disorders. As high as 85.0% of emergency nurses reported experiencing at least one incident of workplace violence in the past year, with psychological violence at 84.4% and physical violence at 61.3%—both rates far exceeding the national averages reported in other departments or regions (31). These figures are also higher than findings from studies conducted in Taiwan (49.6%) and Hong Kong (44.6%) (32, 33). This indicates that the emergency department (ED), as one of the most intense and conflict-prone environments in healthcare, has become a “high-risk zone” for violence against medical personnel (34). The discrepancies may be attributed to differences in research instruments, sampling methods, regional cultural norms, hospital infrastructure, local policies, and training for healthcare workers. Nevertheless, it is undeniable that emergency nurses operate under prolonged high-stress and high-conflict conditions, necessitating the establishment of systematic violence prevention mechanisms and support systems to reduce the incidence and impact of WPV and protect nurses' occupational health.

Further analysis demonstrated a significant positive correlation between total WPV score and total sleep disorder score (*r* = 0.295, *P* < 0.01), indicating that higher exposure to violence is associated with poorer sleep quality. This finding aligns with multiple international studies confirming WPV as a critical psychological stressor affecting nurses' sleep health (19, 20). Notably, in the multiple linear regression model, psychological violence (β = 0.252, *P* < 0.001) emerged as an independent predictor of sleep disorders, with a stronger effect than physical violence. This suggests that although physical aggression is more visible, covert forms of psychological violence—such as verbal abuse, threats, belittlement, and indifference—inflict deeper and more lasting damage on psychological stability (19). Nurses subjected to psychological violence often experience persistent negative emotions such as anger, humiliation, helplessness, and professional doubt, which are difficult to resolve quickly and may evolve into chronic psychological stress (35). Physiologically, prolonged stress can activate the hypothalamic-pituitary-adrenal (HPA) axis, elevating cortisol and adrenaline levels, placing the body in a state of “hyperarousal” suppressing melatonin secretion, disrupting circadian rhythms, and ultimately leading to difficulties in falling asleep, fragmented sleep, and early morning awakening (36, 37). Moreover, negative rumination—repeatedly recalling traumatic events—is common after violent incidents, intensifying anxiety and depression, thereby creating a vicious cycle of “violence → stress → sleep disturbance → emotional deterioration” (38, 39). Evidence shows that healthcare workers exposed to WPV are 2.55 times more likely to suffer from sleep problems than those unexposed (40), and our findings further confirm this association within the emergency nursing population.

In addition to WPV, this study identified several sociodemographic and occupational factors influencing sleep disorders. Marital status showed that married nurses had significantly lower sleep disorder scores than unmarried nurses (*P* = 0.038), a finding that contrasts with some previous studies (41). However, it may reflect the protective role of family support in buffering occupational stress. Strong family relationships can provide emotional comfort, enhance psychological resilience, and promote adaptive coping strategies, thereby mitigating the negative impact of work-related stress on sleep (42). Therefore, occupational health management should emphasize the importance of family and social support systems, encouraging hospitals to implement family-involved psychological interventions.

Regarding professional title, nurses with senior titles exhibited significantly lower sleep disorder scores than junior-level nurses (*P* = 0.009), possibly due to greater clinical experience, stronger communication skills, and higher perceived job control, which may boost confidence in managing patient conflicts and reduce psychological burden. Conversely, nurses with longer work experience (>20 years), longer weekly working hours (41–48 h), and higher night shift frequency (≥9 times/month) faced increased risks of sleep disorders. This suggests that while experience may alleviate some stress, long-term heavy workloads and frequent circadian disruptions remain significant physiological challenges (43). Although shift work ensures continuity of care, it disrupts the body's natural circadian rhythm, leading to sleep homeostasis imbalance and increased risks of insomnia, daytime sleepiness, and fatigue (44). Chronic night shifts may further exacerbate psychological stress by continuously activating the HPA axis, thereby worsening sleep quality (45). Based on these findings, hospital administrators should strengthen violence prevention and response systems, enhance psychological support services, optimize shift scheduling, and reinforce both organizational and familial support to improve the overall occupational health and wellbeing of emergency nurses.

This study employed a cross-sectional design, which, while effective in identifying strong associations between workplace violence and sleep disorders, cannot establish causality. Future longitudinal cohort studies or intervention trials are needed to determine whether psychological violence directly leads to the deterioration of sleep quality. Additionally, all data were collected via self-reported questionnaires, which may introduce recall bias or social desirability bias. Some nurses who experienced severe violence or suffered from significant sleep disturbances might have avoided participation due to psychological avoidance, potentially limiting the representativeness of the sample. Although standardized training and quality control measures were implemented during data collection, such biases cannot be entirely eliminated.

## 5 Conclusion

This study highlights the severe issue of workplace violence faced by emergency nurses in China, with psychological violence identified as a key modifiable factor contributing to sleep disorders. Sleep health is influenced by multiple factors, including marital status, professional title, years of experience, working hours, and night shift frequency. Nursing administrators should strengthen institutional violence prevention systems at the policy level, while also addressing the mental health needs of high-risk groups. By optimizing the work environment, providing psychological support, and implementing flexible management strategies, hospitals can significantly enhance the occupational wellbeing, job satisfaction, and overall health of emergency nurses.

## Data Availability

The raw data supporting the conclusions of this article will be made available by the authors, without undue reservation.
